# Association of different microbes and pathogenic factors in cases of infectious bovine keratoconjunctivitis in cattle from Eastern Kazakhstan

**DOI:** 10.14202/vetworld.2023.1833-1839

**Published:** 2023-09-14

**Authors:** Marat Kuibagarov, Elmira Abdullina, Anara Ryskeldina, Bolat Abdigulov, Asylulan Amirgazin, Alexandr Shevtsov, John A. Angelos

**Affiliations:** 1 National Center for Biotechnology, Astana, 010000, Kazakhstan; 2 Department of Veterinary and Agricultural Management, Shakarim University, Semey, 071412, Kazakhstan; 3 Department of Medicine and Epidemiology, School of Veterinary Medicine, University of California, Davis, California 95616, USA

**Keywords:** bovine herpes virus, infectious bovine keratoconjunctivitis, *Moraxella bovis*, *Moraxella bovoculi*, multiplex real-time polymerase chain reaction, *Mycoplasma bovis*, *Mycoplasma bovoculi*

## Abstract

**Background and Aim:**

Infectious bovine keratoconjunctivitis (IBK) causes a significant economic loss to cattle industries in many countries, including Kazakhstan. Although *Moraxella bovis* is recognized as an etiologic agent of IBK, other bacterial and viral agents have been suspected to play a role in the pathogenesis of this disease. This study aimed to evaluate samples collected from the eyes of IBK-affected cattle in Eastern Kazakhstan at different stages of IBK for the presence of *Mor. bovis*, *Moraxella bovoculi*, *Mycoplasma bovis*, *Mycoplasma bovoculi*, and Bovine Herpes Virus Type 1 (BHV-1) and to characterize *Mor. bovoculi* *pilA* gene sequence diversity from *Mor. bovoculi* positive samples.

**Materials and Methods:**

Individual ocular swabs (n = 168) were collected from cattle that had clinical signs of IBK during the summer of 2022 on farms in the Abay region of Kazakhstan. Eye lesion scores (1, 2, and 3) were assigned depending on the degree of ocular damage. Infectious bovine keratoconjunctivitis-associated organisms were detected using a multiplex real-time polymerase chain reaction assay. The *Mor. bovoculi* *pilA* gene was sequenced from *Mor. bovoculi* positive samples.

**Results:**

*Mycoplasma bovis* and BHV-1 were not detected in any of the collected samples. *Mycoplasma bovoculi* was identified in the majority of samples overall, usually in mixed infection with *Moraxella* spp. *Moraxella bovoculi* was detected in 76.2% of animals and predominated in animals with eye lesion scores 2 and 3. *Mycoplasma bovoculi* was detected only in association with *Mor. bovis* and/or *Mor. bovoculi* in animals with eye lesion scores 2 and 3. *Moraxella bovis* was found in 57.7% of animals and was always identified in association with another organism. Sequencing of the *pilA* gene in 96 samples from *Mor. bovoculi* positive samples identified five PilA groups. The majority belonged to PilA group A. However, three new PilA groups were identified and designated PilA groups N, O, and P.

**Conclusion:**

The results indicate a high prevalence of *Myc. bovoculi* and *Mor. bovoculi* in eyes of cattle with IBK on livestock farms in Eastern Kazakhstan. Additional novel *Mor. bovoculi* PilA groups were identified.

## Introduction

Infectious bovine keratoconjunctivitis (IBK) is a contagious ocular disease of cattle characterized by ulcerative keratitis, corneal edema, and conjunctivitis. This disease is the most common eye condition of cattle [1] and occurs worldwide, including in Kazakhstan [2–4]. Although corneal ulcerations in cattle may heal with limited corneal scar formation, it is not uncommon for IBK to result in complete or partial loss of vision [5]. Infectious bovine keratoconjunctivitis also occurs in other livestock [6] and wild animals [7].

The causative agent of IBK has historically been cited as *Moraxella bovis* [8, 9]; however, more recent studies have identified *Moraxella bovoculi* as the most frequently isolated *Moraxella* from eyes of IBK-affected cattle [10]. Although these observations have suggested that there may be a role for other *Moraxella* spp. in the pathology of this disease [11], published studies to date have not proven a causal association between *Mor. bovoculi* and IBK [12]. The presence of *Mycoplasma* spp. in IBK-affected cattle has also been reported [13–15]. In most IBK cases determined to have a *Mycoplasma* spp., *Mycoplasma bovoculi* is reported [16, 17]. Attempts have been made to clarify the role of *Mycoplasma* spp. in IBK pathogenesis [18]. However, the role of Mycoplasmas in the course of naturally occurring IBK remains poorly understood. Investigations of associations between IBK and Bovine Herpes Virus Type 1 (BHV-1), the causative agent of infectious bovine rhinotracheitis (IBR), have also been investigated and have determined that BHV-1 may be a predisposing factor in IBK [19, 20].

This study was undertaken to characterize microbes associated with clinical cases of IBK in cattle from eastern Kazakhstan using a multiplex real-time polymerase chain reaction (PCR) assay that was developed to identify *Moraxella* spp., *Mycoplasma* spp., and BHV-1. An additional aim of this study was to identify and characterize a *Mor. bovoculi* pilin protein (PilA) in *Mor. bovoculi* positive samples.

## Materials and Methods

### Ethical approval

This study was approved by the Local Ethics Committee of the Kazakh National Center for Biotechnology (Protocol #4 dated September 08, 2020). Cattle owners provided consent for sampling, and no animals were harmed during the sampling process.

### Study period and location

**Figure-1:**
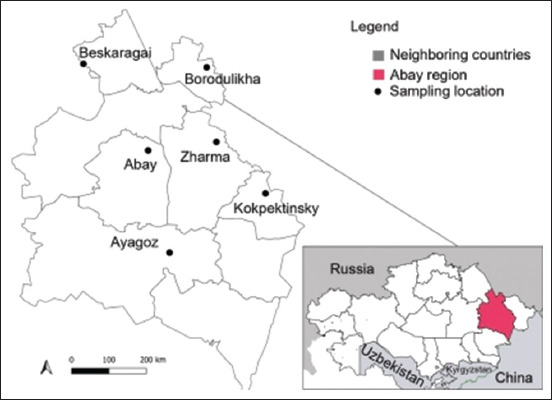
Sampling map [Source: QGIS 3.10].

Sampling of IBK-affected cattle was conducted during July and August 2022 on six farms in six districts of the Abay region of Kazakhstan: Zharma, Kokpektinsky, Borodulikha, Abay, Beskaragai, and Ayagoz. The Abay region is located in the eastern part of Kazakhstan (50°24’40′′N, 80°13′39′′E) (Figure-1). It is dominated by desert steppe and sandy soil and has a continental climate characterized by large daily and seasonal temperature fluctuations; these features contribute to windy and dusty conditions that are characteristic of this area.

### Animals sampled

Sampled animals (n = 168) were not previously vaccinated against IBK, IBR, or Mycoplasma, were under 1 year of age, and had no history of previous antibiotic treatment for IBK. At the time of sample collection, the degree of ocular damage was scored 1, 2, or 3 based on the presence of IBK-associated clinical signs as follows: score 1 (slight corneal edema, lacrimation, blepharitis, and blepharospasm); score 2 (corneal edema with visible defect in corneal surface suggestive of corneal ulceration up to 12 mm in diameter with or without evidence of corneal neovascularization); and score 3 (evidence of corneal perforation with deformation of the eyeball [keratoconus]).

### DNA extraction

Ocular swabs were collected from the edges of abnormally appearing corneal defects and the subconjunctival fornix using sterile cotton swabs. Following sample collection swabs were placed in a test tube containing a lysing solution with chaotropic agents (DNA/RNA-C-factor extraction kit; LLC “Vet factor”, Russia, Moscow, cat. No. EDR001-VET). Samples were transported to the research laboratory within 48 h at 8°C. Further, stages of DNA extraction were performed under laboratory conditions according to instructions provided by the kit manufacturer. The concentration of DNA was determined spectrophotometrically using a NanoDrop1000 (Thermo Fisher, Wilmington, USA) spectrophotometer; extracted DNA was stored at −20°C.

### Positive control samples

Bacteria that had been isolated from cattle with IBK in different regions of Kazakhstan during 2018–2021 and that were identified using matrix-assisted laser desorption/ionization time-of-flight mass spectrometry as either *Mor. bovis*, *Mor. bovoculi*, or *Myc. bovoculi* were used as the source of positive control DNA. *Moraxella bovis* and *Mor. bovoculi* that had been stored frozen in trypticase soy broth with 40% glycerol at −80°C temperature were thawed and cultured on Columbia agar with 5% bovine serum for 20–24 h at 37°C. *Mycoplasma bovoculi* that had been stored frozen in trypticase soy broth with 40% glycerol at −80°C temperature were thawed and cultured on PPLO agar base (TM 233, TM Media, India) with *Mycoplasma* Enrichment Supplement (TS 052, TM Media) at 37°C in the presence of 10% CO_2_. Positive control genomic DNA was isolated from these samples using a commercial kit (QIAamp DNA Mini Kit, Qiagen. Hilden, Germany).

Positive control *Myc. bovis* DNA was artificially synthesized using PCR-based accurate synthesis of long DNA sequences as previously described by Xiong *et al*. [21]. The synthesized sequences were cloned into the pGEM-T plasmid using the Promega pGEM-T Easy cloning kit. (Promega, Madison, USA). The resulting ligation mixtures were used for the subsequent chemical transformation of competent *Escherichia coli* DH5α cells. Five clones were cultured in 7 mL of Luria Broth medium and isolated (Wizard® SV 96 and SV 9600 Plasmid DNA Purification Systems, Promega). The cloned sequence was verified by direct sequencing; a clone without errors in the primer and probe annealing sites was used as a positive control. Positive control BHV-1 DNA was synthesized using the procedure described above for *Myc. bovis*.

### Polymerase chain reaction testing; sequencing of *Mor. bovoculi pilA* gene

To detect *Mor. bovis*, *Mor. bovoculi*, *Myc. bovis*, *Myc. bovoculi*, and BHV-1, the multiplex real-time PCR assay proposed by Zheng *et al*. [22] was used with modifications. The distribution of primers among the mixes corresponded to the described protocol; 6-carboxyl-X-rhodamine, 4-5-dichloro carboxyfluorescein, and carboxyfluorescein were used as sample stains. The volume of the reaction mixture was 25 μL and included 12.5 μL of 2× BioMaster HS-qHCR (OOO Biolabmix, MH020-2040, Novosibirsk), 1.5 μL of each primer and probe at a concentration of 10 pmoL/μL, 5 μL of isolated DNA, and water to reach a final reaction volume of 25 μL. Polymerase chain reaction was performed on a CFX96 Touch (BioRad, Hercules, CA) using the following assay conditions: initial denaturation for 5 min at 95°C followed by 45 denaturation cycles for 15 s at 95°C and combined annealing and elongation for 60 s at 60°C.

To assess the genetic diversity of *Mor. bovoculi*-encoded structural pilin (*pilA*) genes, multiplex real-time PCR samples that were positive for *Mor. bovoculi* were amplified with *pilA* primers (Mbovoc_Pilin_Dn (5’-GTGGGGTTACATAAATATAAAGA-3’) and Mbovoc_Pilin_Up3 (5’-GATTAATCAAACCTTCAAACAC-3’)) [23]. These primers allow the amplification of a full-length *pilA* gene. The efficiency of amplification was evaluated using gel electrophoresis. Polymerase chain reaction products were purified from unbound primers using an enzymatic method using Exonuclease I (Thermo Scientific™. Waltham, Massachusetts, USA) and Shrimp Alkaline Phosphatase (Applied Biosystems™. Vilnius, Lithuania) as described by Werle *et al*. [24] and sequenced with the above reaction primers using the BigDye^®^ Terminator v3.1 Cycle Sequencing Kit (Applied Biosystems. Foster City, USA) according to the manufacturer’s instructions, followed by separation of the fragments using an automated genetic analyzer (3730xl DNA Analyzer; Applied Biosystems). Nucleotide sequences were analyzed with SeqMan software (DNASTAR, Inc. https://www.dnastar.com/software/lasergene/seqman-ngen/. The deduced amino acid sequences obtained from *pilA* amplicons were compared to sequences in GenBank using the Basic Local Alignment Search Tool algorithm, and phylogenetic trees were built based on comparisons with these sequences. PilA-deduced amino acid sequences were also compared to previously published by Kuibagarov *et al*. [3] and Angelos *et al*. [23] PilA sequences for *Mor. bovoculi*.

Evolutionary analyses were conducted in MEGA X (www.megasoftware.net) [25]. The evolutionary history of the deduced amino acid sequences for PilA was inferred using the neighbor-joining method [26]. The optimal tree is shown. The percentage of replicate trees in which the associated taxa clustered together in the bootstrap test (1000 replicates) is shown next to the branches [27]. The tree was drawn to scale, with branch lengths in the same units as those of the evolutionary distances used to infer the phylogenetic tree. The evolutionary distances were computed using the Jones-Taylor-Thornton matrix-based method [28] and are in the units of the number of amino acid substitutions per site. The rate variation among sites was modeled with a gamma distribution (shape parameter = 5). The evolutionary history of the nucleotide sequence was inferred using the maximum likelihood method and Kimura 2-parameter model [29]. The tree with the highest log likelihood (−1613.03) is shown. The percentage of trees in which the associated taxa clustered together is shown next to the branches. Initial tree(s) for the heuristic search were obtained automatically by applying neighbor-joining and BioNJ algorithms to a matrix of pairwise distances estimated using the maximum composite likelihood approach and then selecting the topology with superior log likelihood value. A discrete Gamma distribution was used to model evolutionary rate differences among sites (5 categories [+*G*, parameter = 0.4971]). The rate variation model allowed for some sites to be evolutionarily invariable ([+*I*], 38.55% sites).

### Statistical analysis

The data were summarized using descriptive statistics.

## Results

### Results of multiplex real-time PCR assay

Of the 168 samples that were examined for this study, 138 (82.1%) were collected from animals with clinical signs of IBK corresponding to score 1; 24 (14.3%) to score 2; and 6 (3.6%) to score 3 (Table-1). No *Myc. bovis* or BHV-1 DNA were detected in the 168 DNA samples by multiplex real-time PCR. In 76 (45.2%) animals, *Myc. bovoculi*, *Mor. bovoculi*, and *Mor. bovis* were simultaneously identified. In 34 (20.2%) animals, *Myc. bovoculi* and *Mor. bovoculi* occurred simultaneously. In 10 (6%) cases, a combination of *Myc. bovoculi* and *Mor. bovis* was identified, and in 11 (6.6%) samples, a combination of *Mor. bovoculi* and *Mor. bovis* was found. The presence of only *Mor. bovoculi* was determined in 7 (4.2%) and only *Myc. bovoculi* in 19 (11.3%) samples. In all cases, *Mor. bovis* was identified only in association with another organism. In 11 animals, none of the five studied microorganisms were identified; all of these occurred in animals with score 1 lesions. Animals with clinical signs corresponding to score 2 had the highest percentage of all three organisms (*Myc. bovoculi*, *Mor. bovoculi*, and *Mor. bovis*; Table-1).

**Table-1:** Organisms identified.

Microorganisms identified	Number of tested animals (%)	Clinical severity score		

		Score 1 (%)	Score 2 (%)	Score 3 (%)
*Myc. bovoculi + Mor. bovoculi + Mor. bovis*	76 (45.2)	58 (42.0)	14 (58.3)	4 (66.7)
*Myc. bovoculi + Mor. bovoculi*	34 (20.2)	27 (19.6)	5 (20.8)	2 (33.3)
*Myc. bovoculi + Mor. bovis*	10 (6.0)	8 (5.8)	2 (8.3)	0
*Mor. bovoculi + Mor. bovis*	11 (6.6)	10 (7.2)	1 (4.2)	0
*Myc. bovoculi*	19 (11.3)	19 (13.8)	0	
*Mor. bovoculi*	7 (4.2)	5 (3.6)	2 (8.3)	
*Mor. bovis*	0	0	0	0
None identified	11 (6.5)	11 (8.0)	0	0
Total	168	138 (82.1)	24 (14.3)	6 (3.6)
Total samples with *Myc. bovoculi*	139 (82.7)	112 (66.7)	21 (87.5)	6 (100)
Total samples with *Mor. bovoculi*	128 (76.2)	100 (59.5)	22 (91.7)	6 (100)
Total samples with *Mor. bovis*	97 (57.7)	76 (45.2)	17 (70.8)	4 (66.7)

*Mor. bovoculi: Moraxella bovoculi, Mor. bovis: Moraxella bovis Myc. bovis*=*Mycoplasma bovis, Myc. bovoculi*=*Mycoplasma bovoculi*

### Variability of PilA protein in *Mor. bovoculi*

**Figure-2:**
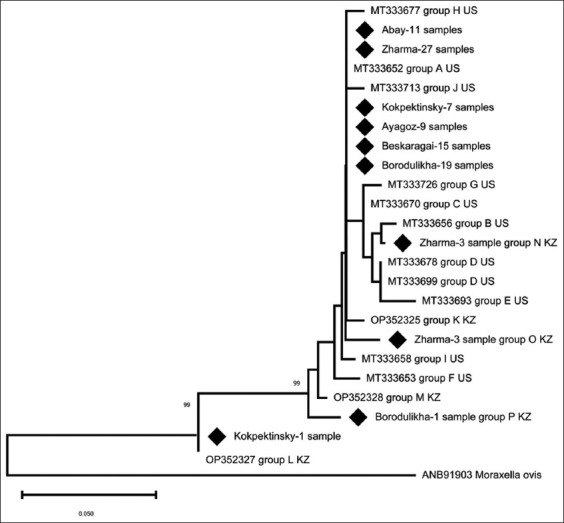
Phylogeny based on the deduced amino acid sequence of the *pilA* gene of *Moraxella bovoculi* determined in samples. The numbers indicate the number of specimens with a given PilA group in a certain district.

A total of 96 of the 128 samples in which *Mor. bovoculi* was identified were amplified using primers specific for *pilA*. In these 96 *Mor. bovoculi* positive samples, a total of 5 PilA groups were identified based on the deduced amino acid sequence for amplified *pilA* genes (Figure-2); numbers indicate the number of specimens with a given PilA group in a certain district. The previously identified A and L groups were found in 88 (91.7%) and 1 (1%) sample, respectively (Table-2). The remaining three groups were not previously reported, were designated N, O, and P groups, and included 3, 3, and 1 samples, respectively.

**Table-2:** Amino acid substitutions identified in *Moraxella bovoculi* PilA^a^.

PilA group designation	Number (%) identified in this study^b^	Position regarding the sequence PilA group A (MT333652)															

		3	16	26	33	61	62	64	77	81	84	90	98	130	131	133	143
A	88 (91.7)	T	I	A	R	S	N	T	Q	V	A	P	I	G	Q	V	D
B	0	•^c^	V	•	•	N	•	•	•	•	V	•	•	•	•	•	•
C	0	•	•	•	•	N	•	•	•	•	•	•	•	•	•	•	•
D	0	•	•	•	•	N	•	•	•	•	V	•	•	•	•	•	•
E	0	•	•	•	•	N	•	A	K	•	V	•	•	•	•	•	•
F	0	•	•	•	•	•	S	•	•	P	•	•	•	•	•	•	•
G	0	•	•	•	•	N	•	•	•	•	•	S	•	•	•	•	•
H	0	•	•	T	•	•	•	•	•	•	•	•	•	•	•	•	•
I	0	I	•	•	•	•	•	•	•	•	•	•	•	S	•	•	•
K	0	•	•	•	•	•	•	•	•	•	•	•	V	•	•	•	•
L	1 (1)	•	•	•	*^d^												
M	0	•	•	•	•	•	•	•	•	•	•	•	•	D	K	•	•
N^e^ OQ835558	3 (3.1)	•	V	•	•	N	•	•	•	•	•	•	•	•	•	•	•
O^e^ OQ835559	3 (3.1)	•	•	•	•	•	•	•	•	•	•	•	•	•	•	I	N
P^e^ OQ835560	1 (1)	•	•	•	•	•	S	•	•	S	•	•	•	D	K	•	•

a Differences from PilA group A are underlined; “•” indicates identity with PilA group A sequence.

b n=number of samples in this study; %=percentage based on the 96 of 128 *Moraxella bovoculi* positive samples for which a *pilA* amplicon was identified.

C Amino acid residue identical to group A.

d Stop codon.

e GenBank accession numbers

On three different farms from three districts (Abay, Beskaragai, and Ayagoz), group A was the only PilA group identified. On a farm from Zharma district, 27 samples had a deduced PilA amino acid sequence that corresponded to group A. However, three samples each did not correspond to previously reported PilA groups and were designated PilA groups O and N. On one farm in the Borodulikha district, 19 accessions were assigned to group A. However, 1 accession did not match a previously characterized PilA group and was designated PilA group P. In the Kokpektinsky district farm, seven samples were assigned to PilA group A and one to PilA group L. The new PilA groups N, O, and P differ from 2 to 4 amino acid residues (Table-2).

Nucleotide sequences of *pilA* genes from this study have been deposited in GenBank (accession numbers OQ835558 – OQ835560).

## Discussion

The aim of this study was to analyze animals with different clinical manifestations of IBK for the presence of *Mor. bovis*, *Mor. bovoculi*, *Myc. bovis*, *Myc. bovoculi*, and BHV-1, and to establish if any relationship existed between the detected pathogens and clinical manifestations of disease in cattle herds from Eastern Kazakhstan. Infectious bovine keratoconjunctivitis is present in cattle from many parts of the world, including Kazakhstan [1]. Although *Mor. bovis* has traditionally been considered as the causative agent of IBK [8, 9], other organisms including *Mor. bovoculi*, *Mycoplasma* spp., especially *Myc. bovis*, and *Myc. bovoculi*, *Chlamydia* spp., *Listeria monocytogenes*, *Ureaplasma* spp., and some viruses, including BHV [30] have also been identified in cattle with eye lesions that resemble IBK and these might also play a role in disease pathogenesis.

*Mycoplasma bovis* and BHV-1 were not detected in any of the samples collected for this study, and apparently, the prevalence of these pathogens in animals from the farms sampled for this study is apparently low. In the World Animal Health Information System (WAHIS) for the period July 2021–June 2023, seven outbreaks of IBR involving 131 animals (causative agent: BHV-1) (https://wahis.woah.org/) were registered in Kazakhstan. There are no data on the prevalence of *Myc. bovis* in Kazakhstan, although this organism has been identified in neighboring Russia and China [31–33], and it is probably more commonly identified as the causative agent of caseonecrotic pneumonia, mastitis, and arthritis [31].

*Mycoplasma bovoculi* was the most common species identified in the analyzed samples of this study (82.7%). In score 1 animals, *Myc. bovoculi* was identified in 81.2% of samples, including 13.8% of samples in which it was identified without *Mor. bovis* or *Mor. bovoculi*. In score 2 and 3 animals, the positive percentage for *Myc. bovoculi* reached 87.5% and 100%. A previous study by Hanzlicek and Bai [34] that utilized PCR to test eye swab samples showed the presence of *Myc. bovoculi* in 99% of IBK samples. Although *Myc. bovoculi* has been identified in calves with characteristic clinical signs of IBK in the absence of *Mor. bovis* or *Mor. bovoculi* [15], the exact role of this pathogen in ocular pathology associated with IBK has not been determined. However, some studies have suggested that *Mycoplasma* spp. may predispose to IBK [35, 36].

Similar to previous studies, in this study, *Mor. bovoculi* was detected in a high proportion of sampled animals. In the studies of Schnee *et al*. [35], although *Mor. bovoculi* prevalence was relatively low (20%), it was the only pathogen whose presence correlated with the occurrence of clinical cases of IBK. An interesting observation was the detection of *Mor. bovoculi* in animals in this study without *Mor. bovis*. While this observation might seem to support a case for *Mor. bovoculi* having a role in IBK pathogenesis, studies published thus far have not demonstrated that *Mor. bovoculi* (ATCC strain BAA-1259) can cause keratoconjunctivitis in experimentally infected animals [12]. In most of the animals sampled in this study, multiple bacterial species could be identified. Previous studies by Bartenslager *et al*. [37] have demonstrated a rich ocular microbiome in cattle. However, whether or not interactions between organisms present in the eyes of cattle allow different populations of bacteria to affect the host in some way that directly leads to ocular injury remains poorly understood. Nevertheless, our PCR testing also indicated the dominance of *Mor. bovoculi* in IBK samples, which were detected in ~76%, and *Mor. bovis* in ~60%, while *Mor. bovoculi* was detected in 24.4% of the samples separately or together with *Myc. bovoculi*. The apparent genetic diversity, presence of virulence factors, and potential for recombination events among *Moraxella* spp. associated with eyes of cattle with IBK [38, 39] underscores the fact that more research is needed to elucidate the role of *Moraxella* spp. in IBK. That a second genotype of *Mor. bovis* [40] and a new species of *Moraxella* (suggested name *Moraxella oculobovii* were also recently reported [41], further illustrating a need for more research into this important disease of cattle.

Among the 96 *Mor. bovoculi* samples for which a *pilA* amplicon was present, we identified 5 *pilA* sequences corresponding to five different PilA-deduced amino acid sequences. Three of these (designated PilA groups N, O, and P) have not previously been reported. The diversity of *pilA* found in this study indicates the existence of genetic polymorphism in *pilA* of *Mor. bovoculi* circulating in Kazakhstan. Subsequent genome-wide analysis of strains in different regions will allow a better assessment of genetic diversity and any possible associations with specific clinical manifestations of IBK. Our study demonstrates a significant dominance of PilA group A, which accounted for more than 91% of our isolates. A possible reason for the high prevalence of group A in this study is the limited region and sampling period, as well as the relatively recent registration of IBK in the surveyed farms (reported since 2016). However, this was also the most common PilA group previously reported by Kuibagarov *et al*. [3] and Angelos *et al*. [23] among *Mor. bovoculi* in the United States and Kazakhstan, although in those studies, the proportion did not exceed 68%. Despite the identification of 15 PilA groups reported in *Mor. bovoculi* from the USA and Kazakhstan, it is worth noting the high degree of conservation of PilA in *Mor. bovoculi* as previously reported by Angelos *et al*. [23]. The maximum number of amino acid residues that the novel PilA sequences from this study differed from PilA group A was 4 out of 152 amino acid residues. An exception is the L group, in which a truncated protein consisting of 32 amino acid residues was observed. This group was found in a score 2 animal. *Moraxella bovis* and *Myc. bovoculi* were also identified in this animal. However, these two organisms were also isolated from other *Mor. bovoculi* positive score 2 animals for which a full-length *pilA* amplicon was identified, so the significance of the truncated PilA sequence is difficult to assess from our data. Subsequent genome-wide analysis of strains in different regions will allow a better assessment of the genetic diversity of *Mor. bovoculi* in Kazakhstan and any possible associations between such diversity and the clinical manifestations of IBK.

## Conclusion

This is the first study on species and PilA diversity of microorganisms associated with IBK in cattle in Eastern Kazakhstan. The results indicate a high prevalence of IBK in cattle on livestock farms in this region associated with *Myc. bovoculi* and *Mor. bovoculi*, as well as the presence of additional novel PilA groups in *Mor. bovoculi*.

## Authors’ Contributions

MK: Designed and supervised the experiments and drafted sections of the original manuscript. AR, BA, AA: Multiplex and conventional PCR analysis, sequencing, and phylogenetic tree analyses. EA: Sample and clinical signs analysis. AS: Molecular investigations and data analysis, and drafted and revised the manuscript. JAA: Data interpretation, critical review, drafted sections of the original manuscript and edited and revised the manuscript. All authors have read, reviewed, and approved the final manuscript.
